# Industrial Biotechnology Conservation Processes: Similarities with Natural Long-Term Preservation of Biological Organisms

**DOI:** 10.3390/biotech12010015

**Published:** 2023-01-31

**Authors:** Alexis Laurent, Corinne Scaletta, Philippe Abdel-Sayed, Wassim Raffoul, Nathalie Hirt-Burri, Lee Ann Applegate

**Affiliations:** 1 Regenerative Therapy Unit, Lausanne University Hospital, University of Lausanne, CH-1066 Epalinges, Switzerland; 2 Faculty of Biology and Medicine, University of Lausanne, CH-1015 Lausanne, Switzerland; 3 Applied Research Department, LAM Biotechnologies SA, CH-1066 Epalinges, Switzerland; 4 Manufacturing Department, TEC-PHARMA SA, CH-1038 Bercher, Switzerland; 5 DLL Bioengineering, STI School of Engineering, Ecole Polytechnique Fédérale de Lausanne, CH-1015 Lausanne, Switzerland; 6 Lausanne Burn Center, Lausanne University Hospital, University of Lausanne, CH-1011 Lausanne, Switzerland; 7 Plastic, Reconstructive, and Hand Surgery Service, Lausanne University Hospital, University of Lausanne, CH-1011 Lausanne, Switzerland; 8 Center for Applied Biotechnology and Molecular Medicine, University of Zurich, CH-8057 Zurich, Switzerland

**Keywords:** anabiosis, biotechnology, biological organisms, cell therapies, cryopreservation, dormancy, freeze-drying, lyophilization, phase transitions, water

## Abstract

Cryopreservation and lyophilization processes are widely used for conservation purposes in the pharmaceutical, biotechnological, and food industries or in medical transplantation. Such processes deal with extremely low temperatures (e.g., −196 °C) and multiple physical states of water, a universal and essential molecule for many biological lifeforms. This study firstly considers the controlled laboratory/industrial artificial conditions used to favor specific water phase transitions during cellular material cryopreservation and lyophilization under the Swiss progenitor cell transplantation program. Both biotechnological tools are successfully used for the long-term storage of biological samples and products, with reversible quasi-arrest of metabolic activities (e.g., cryogenic storage in liquid nitrogen). Secondly, similarities are outlined between such artificial localized environment modifications and some natural ecological niches known to favor metabolic rate modifications (e.g., cryptobiosis) in biological organisms. Specifically, examples of survival to extreme physical parameters by small multi-cellular animals (e.g., tardigrades) are discussed, opening further considerations about the possibility to reversibly slow or temporarily arrest the metabolic activity rates of defined complex organisms in controlled conditions. Key examples of biological organism adaptation capabilities to extreme environmental parameters finally enabled a discussion about the emergence of early primordial biological lifeforms, from natural biotechnology and evolutionary points of view. Overall, the provided examples/similarities confirm the interest in further transposing natural processes and phenomena to controlled laboratory settings with the ultimate goal of gaining better control and modulation capacities over the metabolic activities of complex biological organisms.

## 1. Introduction

It is well established since the early days of the natural sciences that water is a ubiquitous and necessary reagent for the formation and the sustenance of many organic-based biological lifeforms [1,2,3,4]. According to evolutionary principles in primordial ecosystems (e.g., the early appearance of primitive biological lifeforms), close considerations of the responses and adaptations of a defined entity to the surrounding environment are necessary [2,4]. Therein, the influence of local temperature, atmospheric pressure, and radiation exposure constitutes a key modulator of the various physical states of water as well as water phase transitions [3,4].

At a molecular level, the careful and formidably complex equilibrium which characterizes metabolic activity in biological lifeforms has first and foremost adapted to exist and persist despite harsh localized environmental constraints [1,4]. Such external influences and drivers incurred iterative and appropriate evolutionary steps. Specifically, low temperatures and low-pressure environments have harbored and shaped water-containing biological organism structures and their energy exchange parameters. Within this necessary adaptation to physical changes in the surrounding environment, an intrinsic capacity to modify the metabolic activity rate within a defined system has appeared and persisted. Further down the evolutionary road and billions of years later, such natural metabolic rate modifications may be observed in numerous forms and functions, which non-exhaustively include hibernation, seeding and sporulation, or cryptobiosis [5,6,7,8]. 

Specifically, many biological lifeforms have naturally evolved and survived partly based on their intrinsic capacity to modulate their responses to environmental physical parameter modifications [6,7,8]. Therefore, indirect and partial insights may be gained about the early stages of the existence of biological lifeforms by the close consideration of defined and controlled biotechnological conservation processes (e.g., cell and tissue cryopreservation or lyophilization). In such artificial examples, the localized environmental physical parameters are manipulated. Therein, specified physical process parameters are known to majorly impact several key and critical attributes of the biological samples under treatment [9,10,11,12,13,14]. Cryopreservation and lyophilization deal with extremely low temperatures, multiple physical states of water, and multiple water phase transition types (e.g., freezing, thawing, sublimation). Both conservation processes have been extensively and successfully employed in biotechnological, pharmaceutical, or food industries and in human medical transplantation workflows [13,14,15,16,17,18]. Specifically, notable biotechnological applications comprise the successful long-term storage of biological samples and products, with a reversible quasi-arrest of metabolic activities (e.g., cryogenic cell storage in liquid nitrogen). The historical background of large-scale cryopreservation and lyophilization processes covers most of the twentieth century, as diverse industrial and scientific communities have used these principles for optimized material processing or for enhanced conservation purposes [9,10]. The current status of research in the field of cryopreservation or lyopreservation is mainly oriented toward the major goal of successfully preserving whole mammalian organs for transplantation purposes [12,14]. Therefore, many bioinspired or biomimetic approaches and solutions may be further investigated and employed, for process-based technical optimization. 

**Figure 1:**
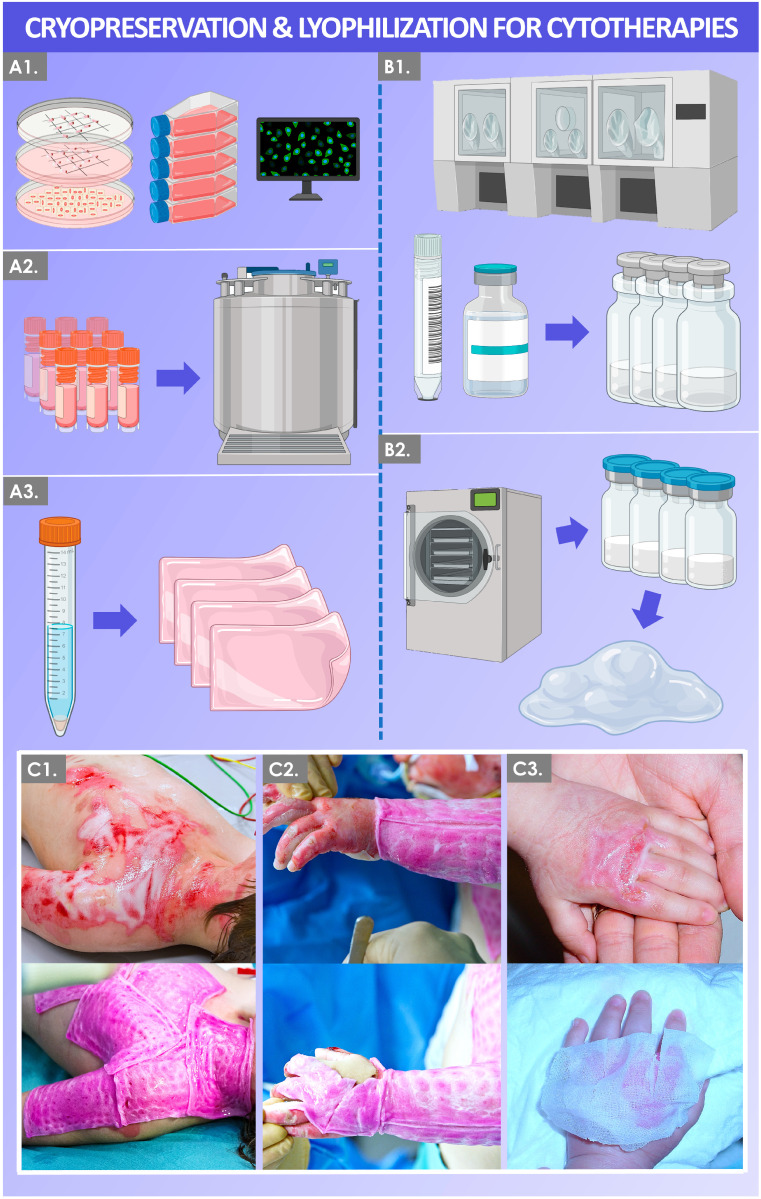
Schematic and illustrative workflow of cryopreservation (**A1**–**A3**) and lyophilization (**B1**,**B2**) processes used in the manufacture of topical cytotherapies or derivatives for cutaneous wound management (**C1**–**C3**) under the Swiss progenitor cell transplantation program. (**A1**) Following in vitro primary progenitor cell type (e.g., FE002-SK2 cells, FE002-Ten cells, FE002-Cart cells) establishment, extensive characterization, and validation work is performed to qualify the standardized biological materials in view of cytotherapeutic use. (**A2**) Multi-tiered in vitro cell banking is used for the manufacture and the long-term storage of the primary progenitor cellular materials. (**A3**) Following medical prescription, viable progenitor cells are initiated from cryostorage and are used to prepare standardized transplant products for cutaneous wound management (e.g., progenitor biological bandages). (**B1**) To resolve several logistical and stability issues, cellular materials and cell-free derivatives are used to formulate appropriate liquid preparations containing cryoprotectants and/or lyoprotectants. (**B2**) The cellular extracts and/or derivatives are then stabilized by two-step lyophilization processing, which enables long-term storage and reconstitution in a wide variety of appropriate pharmaceutical preparations for clinical delivery (e.g., hyaluronan-based hydrogels, oil-in-water creams or ointments). (**C1**–**C3**) Documented illustrative examples of pediatric burn patient wounds treated with cultured and cryopreserved cells formulated into progenitor biological bandages (PBB) in the Lausanne burn center (Lausanne, Switzerland). Previous studies have shown that PBB cytotherapeutic treatment of the burn wounds enables enhanced quality of cutaneous repair, with restored structure and function and diminished rates of complication as compared to the use of standard wound dressings. Modified and adapted with permission [11,12,15].

This study firstly considers the controlled laboratory/industrial artificial conditions used during cryopreservation and lyophilization of cellular materials within regenerative medicine workflows, based on 30 years of experience by the authors. In particular, the use of both biotechnological conservation tools was briefly described for the investigational and translational research performed under the Swiss progenitor cell transplantation program around primary progenitor cytotherapies and derivatives (Figure 1) [11,12]. Therein, initial clinical work and reports by the authors were related to the topical cytotherapeutic use of viable primary progenitor dermal fibroblasts for the management of burn wounds [19,20]. Such primary cells were manufactured and stored in multi-tiered cell bank systems, as classically used in biotechnological processing workflows [11,12]. At present, progenitor biological bandages (PBB, i.e., viable cells on a bioresorbable collagen sheet scaffold) are clinically used to favor enhanced quality of cutaneous healing in Swiss burn patients [11,21]. Beneficial effects for cutaneous healing are procured by the cells notably via stimulation of the wound bed (i.e., promotion of patient cell proliferation and migration) and local delivery of growth factors, cytokines, and extracellular matrix components [21]. Despite highly encouraging clinical results obtained for diverse groups of patients, technical limitations of therapeutic cell lot cryopreservation were outlined, such as burdensome logistical and transport parameters for delivery of finished products to the clinic [21]. Therefore, alternative therapeutic material formulation and processing methods were then experimentally investigated (e.g., use of lyophilized cellular preparations and stabilized derivatives), for optimization of regulatory processes and clinical pathways [12].

This study secondly outlined similarities between the controlled environments of laboratory or industrial cryopreservation/lyophilization and some natural ecological niches known to favor metabolic rate modifications (e.g., cryptobiosis, sporulation) in selected biological lifeforms [1,2,4,22]. Overall, the provided examples confirm the interest of further transposing natural processes and biological phenomena to controlled laboratory settings (i.e., use of bioinspired conservation processes), with the ultimate goal of gaining better control and modulation capacities over the metabolic activities of complex biological organisms.

## 2. Cryopreservation of Biological Tissues and Cells: Principles and Application in Cytotherapies

Cryopreservation with cryogenic storage of biological tissues and cells allows for significant slowing of metabolic rates and may effectively protect valuable samples from time-related deterioration [23,24,25,26,27]. Therefore, this method uses extremely low temperatures (e.g., −160 °C to −196 °C) to enable the extensive prolongation of the existence of metabolic activity and specified organism functions within the considered sample, with a transiently altered/stabilized state. Therein, due to the designed slowing down of chemical and enzymatic reactions at cryogenic temperatures, the preserved system is placed in a reversible state of quasi “suspended animation” during storage. This technical possibility has been extensively leveraged in human medicine practices in order to preserve high-value biological samples from the effects of time [16,23,24,25].

Successful cryopreservation of biological samples such as mammalian cell suspensions is achieved with the use of appropriate cryoprotectants (i.e., usually small molecules and polymers), which act to stabilize the system during temperature variations and physical phase changes (i.e., freezing) [16]. Cryoprotectants used in the biotechnology field usually act by increasing the intracellular solute concentration, which protects the cells from ice crystal formation and freezing damages by lowering the glass transition temperature of the system [16]. Therefore, cryoprotectants must be able to enter the cell and should not cause direct cytotoxicity. In order to limit such potential toxicity, cryoprotectant combinations may be employed. Classically, glycols such as glycerol and dimethyl sulfoxide (DMSO) may be used for highly efficient preservation of cells and biological samples in liquid nitrogen [16,23,25]. On the other hand, frequent natural examples of cryoprotectants are sugars and polyols (e.g., glucose, trehalose) or antifreeze proteins [7]. Interestingly, while diverse natural examples of successful small organism freezing tolerance and preservation exist (e.g., frogs, salamanders), considerable challenges still exist in the organ transplantation field, wherein whole-organ preservation in frozen states remains highly complex [13,14,16]. 

As many biological entities exist in an amorphous form (i.e., relatively low glass transition points) once frozen, temperatures close to −196 °C (i.e., the boiling point of liquid nitrogen) are considered to be necessary to substantially slow down almost all metabolic activity [20,22]. With regard to cytotherapeutic product cryopreservation, the physical parameter (i.e., temperature within the freezing system) modifications and the related rates of modification used to bring the samples from ambient temperatures down to cryogenic temperature levels are crucial (i.e., rate of sample freezing in °C/min before transfer to liquid nitrogen storage tanks, Figure 1A2) [16,23,28,29,30]. It is well established that the control level over the sample freezing and thawing phases mainly dictates the overall effectiveness of the cell cryopreservation process, in terms of structural integrity maintenance and cellular viability recoverability (Table 1).

As concerns the described example of topical allogeneic cytotherapeutic application of the progenitor biological bandages, it should be noted that the cellular active substance is cryogenically preserved as an off-the-freezer source, yet the finished product (i.e., as delivered to the clinic) is not stored in frozen form. Indeed, a short (i.e., 24–48 h) recovery period following cell thawing enables the optimal restoration of both cellular viability and function [21]. This approach is more rapid than the use of autologous cutaneous tissue engineering products (e.g., cultured (dermal-)epidermal autografts) following patient admission, as in vitro cell manufacturing is already performed [21]. For immediate treatment of a burn patient following admission, a ready-to-use wound dressing is necessary, which may be acellular (e.g., Alloderm, Integra, Biobrane) or retrieved from a tissue bank (e.g., cadaveric skin graft) [31]. Interestingly, clinical centers in Belgium and around the world have reported several advantages around the application of frozen or otherwise preserved stratified keratinocyte sheets for burns as a ready-to-use finished cytotherapeutic product [32,33,34,35].

Critical damage to cells and biological samples upon temperature lowering may include extracellular and/or intracellular ice crystal build-up (i.e., mechanical damage to cellular structures), solution effects (i.e., osmotic toxicity), and dehydration (i.e., osmotic toxicity or collapse) (Table 1) [36,37,38,39,40]. Specific material manipulation techniques such as persufflation of solid organs or the addition of various synthetic cryoprotectants may be used to additionally stabilize the samples [39,40,41].

Importantly, the selection of optimal physical parameter modification rates (e.g., −1 °C/minute rate of cooling) proves to be of prime importance to reversibly attain a stable cryogenic state (Table 1). Therein, slow controlled-rate freezing or vitrification (i.e., rapid cooling of optimized viscous and amorphous systems) may efficiently be used at industrial scales prior to long-term liquid nitrogen storage [41,42]. Appropriate methods of sample thawing (i.e., usually with rapid warming) and rinsing are considered as equally important at the time of retrieval and initiation, for optimal cellular recovery (Table 1). Internal experience around the cryopreservation of primary progenitor dermal fibroblasts for topical cytotherapeutic use has indicated that conservative best practices enable to consistently obtain 95 ± 5% cellular viability levels within the first decade of liquid nitrogen storage [11,20,21].

In research and development applications, cryopreservation is mostly employed to conserve defined cell types and cell lines or modified biological derivatives for extended time periods [23,43]. A prime example of successful multi-tiered primary cell banking for industrial applications is the WI-38 cell type, extensively used for over five decades in numerous research and manufacturing settings following a single starting material procurement step [44]. Furthermore, a major interest and advantage of cryopreserving bacterial and fungal strains lies in the avoidance of genetic or phenotypic drifts, possibly occurring in refrigerated storage.

In widespread clinical applications, cryopreservation is successfully used for the conservation of solid organs or blood products and reproductive materials (i.e., oocytes, embryos, ovarian tissues, semen, umbilical tissues) [16,23,28,29,36,45,46]. Therein, sufficient hindsight is currently available for the assessment of the safety and the effectiveness of sperm and embryo cryopreservation, with no reported major increases in birth defects or child development abnormalities [45,46]. While cryogenic processes technically enable indefinite biological material storage, medical practices very rarely comprise the use of cryopreserved sperm after 20 years of preservation or the use of frozen embryos after a decade of storage. As regards cryopreservation of cells for clinical use (i.e., in classical cytotherapies), the available evidence suggests that direct administration following material initiation from storage yields diminished efficacy results, as compared to cells which are allowed to optimally recover in vitro before product formulation [36,47].

Furthermore, while cryopreservation workflows are well-adapted and simply implemented for certain types of cells, many cytotherapeutic materials require extensive conservation process optimization or cannot be effectively cryopreserved altogether. Specifically, extensive hindsight is currently available for the preservation of hematopoietic stem and progenitor cells (HSPC) or mesenchymal stromal/stem cells (MSC) for clinical use [36]. Therein, several challenges and important considerations were outlined around the cryoconservation step of the manufacturing process, which is necessary for regulatory and economic viability of many therapies [36]. In particular, suboptimal conservation processes may drastically impact in vitro manufacturing activities in case of low cell survival or proliferation capacity after thawing of seeding cell lots. Secondly, negative impacts on therapeutic material efficacy may be incurred by cryopreservation, leading to low cellular payload survival, engraftment, and activity in vivo [16,23,36]. While modern advancements in technological tools may enable to optimize the cryoconservation of selected cell therapies, others may require direct use after manufacturing, to guarantee optimal safety and efficacy. By extension, the cryopreservation of biological samples containing various cell types (e.g., tissue engineering products for multi-layer cutaneous replacement) currently appears as highly challenging, as each cell type may behave differently during processing and therefore require specific conditions for successful preservation. 

Within the classical cytotherapeutic applications investigated under the Swiss progenitor cell transplantation program, the use of cryopreservation with the cell banking workflows comprises multi-parameter advantages [11,15]. Firstly, the on-demand availability of cryopreserved allogeneic cellular material stocks reduces the overall therapy manufacturing delays to hours, enabling rapid administration following prescription. Then, the possibility of using extensive multi-tiered cell banking workflows enables optimization of material standardization and safety (i.e., iterative controls and post-production batch release) [12]. Finally, the high stability of the considered cellular materials in cryogenic storage makes it possible to potentially manufacture millions of cytotherapeutic product units following a single primary cell type establishment procedure. In parallel, the extensive shelf-life of cryogenically preserved cellular material lots (i.e., >2 decades for primary progenitor cells) enables us to perform extensive manufacturing campaigns at regular intervals, leading to diminished manufacturing infrastructure use (i.e., the main driver of fixed manufacturing costs) [11,20,21]. 

Overall, the use of appropriate cryopreservation techniques demonstrably leads to rationalization of technical, regulatory, and economic resources in modern allogeneic regenerative medicine workflows [11]. Specifically, the possibility of performing extensive endpoint quality controls on manufactured biological materials (e.g., cryopreserved cytotherapeutic cell lots) enables to reduce risks as compared to rapid testing of fresh cell batches for direct clinical use. This in turn enables to better justify and demonstrate the quality of the intervention from a regulatory standpoint, provided that the cryopreservation phase does not negatively impact critical attributes of the considered cells [21,36]. However, disadvantages are also linked to the use of cryopreservation processes in the manufacturing and distribution of cytotherapies. Indeed, cryogenic storage infrastructures are costly to maintain and logistical workflows requiring liquid nitrogen or dry ice shipping comprise environmental and occupational safety hazard concerns. Such factors should be taken into account in the development of new cell therapies as they limit the number of patients having access to such treatments [21,28,29,30].

## 3. Natural Forms of Environment-Mediated Cryopreservation and Freezing Tolerance 

When considering freezing tolerance of whole biological organisms, relatively few natural examples have been reported to date (e.g., snails, frogs; Figure S2). However, the bioinspired technical possibility of restoring metabolic activity following a defined cryogenic state continuously motivates advocates of lifespan extension by cryonics [48]. Notably, small multicellular organisms such as daphnia, bdelloid rotifers, or tardigrades have been reported to withstand long-term freezing, possibly by producing or storing high levels of trehalose, which acts as a natural cryoprotectant, and by using alternative mechanisms (Figure S2) [8,49,50,51,52]. Larger organisms such as frogs were reported to use urea and polyols to withstand (partial) winter freezing, which is also achieved by some turtles, salamanders, lizards, and snakes [53].

Antifreeze proteins were shown to enable freezing tolerance or freezing avoidance in a number of animals and plants or bacteria and fungi by binding to ice crystals (i.e., by adsorption) under formation. Therefore, such polypeptides are different from solute glycols and sugars in their mechanism of action under freezing temperatures [54,55]. Although modern advances in biotechnology may enable to further use such mechanisms in a bioinspired and gene edition setting, they are considered to be more complex and difficult to apply in simple and large-scale cell cryopreservation applications. Conversely, the metabolic and cryoprotective use of trehalose by many natural species has been studied and applied in laboratory and industrial settings, notably for optimized protein, cell, or biological tissue preservation [7,49]. Overall and despite some differences between the natural and laboratory environments, it may be assessed that sugars and polyol-based substances such as trehalose are very effectively used to procure similar effects of protection against freezing.

Of high interest, very long-term (i.e., >11,700 years) freezing tolerance has been evidenced in natural permafrost environments for microscopic nematodes [56]. This example demonstrates that when optimal environmental conditions are locally created in nature, successful sample transformation and stabilization may enable some form of biological activity restoration, even tens of thousands of years later [56]. Although these extreme examples may be attributed to chance in natural environments (e.g., similarly to the synchronized environmental conditions required for fossil formation) and may be extremely difficult to replicate in controlled laboratory settings, they tangibly demonstrate that the technical possibility exists. 

## 4. Lyophilization: Principles and Application for Temperature-Stabilized Cytotherapeutic Derivatives

Freeze-drying or lyophilization is widely used in controlled laboratory and industrial settings as an effective means of stability enhancement by careful and low-temperature removal of sample water [57,58]. In addition to the liquid to solid water phase transition undergone during sample freezing (i.e., similar to cryopreservation), subsequent water phase transitions are made possible and favored by the creation of specific localized physical conditions (i.e., vacuum creation under low temperatures and controlled energy supply, Table 2) [59,60,61,62,63].

Therefore, high qualitative gains are procured by the use of appropriate freeze-drying methods (i.e., as compared to water evaporation) which enable both water sublimation and desorption to take place without excessive sample heating (Table 2) [60]. However, similar to cryopreservation methods, the sample freezing phase preceding primary drying initiation represents a capital step yielding important impact on the quality of the eventually rehydrated material [62]. From a processing point of view, the argument may be made that the preliminary freezing step is the most important phase of the whole lyophilization process as all the subsequent sample processing steps depend on the exact state of the frozen sample batch at the beginning of primary drying.

From a mechanistic point of view, the resistance of specific biological structures to the removal of water molecules is made possible by the use of lyoprotectants. Therefore, small molecules and polymers which are used in the pharmaceutical industry for formulation of lyophilization batches may act by replacing the water molecules which are removed during processing, thereby limiting the occurrence of damaging structural modifications [9,10,18,58]. Classically, sugar-based polymers (e.g., saccharose, mannitol, dextran) are used as lyoprotectants for drugs, peptides and proteins, or cellular materials [12]. The need for a lyoprotectant may be partially reduced by acting on the rate of freezing of the system, as rapid freezing results in the formation of small ice crystals, which are less detrimental to biological structures than large ice crystals (Table 2). From a formulation point of view, lyoprotectants used in biotechnology (i.e., sugars) are highly similar to those found in desiccation-tolerant small invertebrate animals such as nematodes and tardigrades. Specifically, the use of trehalose as a lyoprotectant in laboratory and industrial settings may be considered as a bioinspired application of desiccation damage avoidance [6,7,8,49,50,51]. Overall, more similarities than differences may be considered when comparing human-controlled and natural processes of lyopreservation from a formulation point of view based on the similar or identical nature of the employed lyoprotective agents (e.g., trehalose). 

Due to sample processing at relatively low temperatures, lyophilized solid objects (e.g., in the food industry) may optimally maintain important attributes such as shape, color, odor, and taste [18]. Similarly, liquid samples (e.g., parenteral pharmaceuticals) formulated with appropriate excipients, cryoprotectants, and lyoprotectants may adopt an optimal physical structure in lyophilized states, characterized by high robustness and stability for prolonged shelf-life [62].

Despite relatively high equipment and operating costs, freeze-drying has been widely adopted in research (i.e., biological strain preservation), medical (i.e., tissue transplant storage or therapeutic product formulation), and industrial fields (e.g., food processing and preservation optimization) [18,35,64,65,66,67,68,69,70,71]. Specific activities or contexts and the large-scale demand for optimized material packaging and transport modalities have historically driven the technical development in the field of lyophilization (e.g., preparation of penicillin or blood products for soldiers, food for astronauts, etc.). Additionally, alternative processing workflows for conservation methods have been successfully applied and have yielded similar results [72,73,74,75].

In the biotechnology field, attaining lyophilized mammalian cell survival and restored function (i.e., similar to cryopreserved cells) post-rehydration is extremely difficult to attain within a standard freeze-drying process [35,64,68]. Despite the maintenance of most cellular structures in optimal drying conditions, the different water phase transitions and water movements during lyophilization processing often prove detrimental to the cells with regard to viability maintenance (Table 2). However, many types of biological derivatives may be efficiently lyophilized and are able to retain appropriate biological or physical/chemical functions, despite viability loss for the cells [12,35,65,66,67,68,69,70,71]. Such results obtained by the authors and others have favored the investigation of cell-derived cell-free biological entities or complexes, which may bare several technical, therapeutic, logistical, or regulatory advantages as compared to classical cytotherapies using fresh living cells, for example (Figure 1) [12,65,71]. This approach has notably enabled progenitor cellular extract long-term storage and reconstitution in a wide variety of appropriate pharmaceutical preparations for investigational clinical use (e.g., hyaluronan-based hydrogels, oil-in-water creams or ointments) under the Swiss progenitor cell transplantation program (Figure 1).

## 5. Natural Cycles of Active Life and Latent Life: Animal, Plant, and Microorganism Dormancy

Numerous natural examples exist and demonstrate the varied mechanisms developed for accommodation to specific environmental conditions (e.g., freezing temperatures, reduced pressures, environmental water availability variations) or to a periodic lack of natural resources [5,6,7,8,76,77]. The function of such natural processes often consists in delaying or synchronizing the physiological development or activity of the organism to varying external parameters such as seasons or climatic particularities in order to maximize fitness [76,77]. Therein, noteworthy examples for processes of periodic dormancy in animals notably comprise hibernation, torpor, estivation, cryptobiosis, brumation, or diapause (Figure S2) [78,79]. 

While many natural dormancy strategies aim to diminish energy expenditure (e.g., hibernation) in defined and cyclic environmental conditions, others are necessary to withstand extreme adverse conditions over long time-periods (e.g., cryptobiosis) [8,78]. Similarly, the control strategies governing the various types of dormancy may be predictive or consequential to environmental factors and external influences (e.g., quiescence) [76,77,78,79].

Dormancy is also well described for plants and may be considered as relatively more important than for animals since plants have limited means of acute adaptation to the environment (i.e., static location, no rapid way to acquire shade or insulation). In addition to the responses to seasonal variations by specific organisms (e.g., vernalization), plant seed dormancy or seed coat dormancy may play preponderant roles in the development and evolution of many species [5,76]. 

Similar tools have been developed by bacteria, which may withstand temperature extremes, desiccation, and antibiotic treatment by forming cysts, endospores, or other states of highly reduced metabolic activity (Figure S2). As a means of survival and dispersal, spores constitute a major role of bacterial or fungal life cycles as well as species evolution and diversity maintenance [77].

As concerns viruses, latency phases may not be strictly assimilated to dormancy, due to the inherent lack of metabolic activity. However, viral latency is also known to constitute a formidable evolutionary drive through the ability to interact with cellular organisms at almost all physiologic and genetic levels [80,81]. 

When considering the robustness and versatility of the adaptation potential of natural multicellular organisms, tardigrades have been studied very extensively [8,82]. Tardigrades, also known as water bears, have been reported to present exceptional resistance and survival capabilities with regards to extreme temperatures, pressures and vacuum, ionizing radiation sources, and anhydrous environments, making them physically and geochemically highly extremotolerant in their natural or cryptobiotic states (Figure S2). The most eloquent example thereof is constituted by their ability to survive in the relative void of space in low Earth orbit [83]. Such resistance in a state of stasis may be the result of cryobiosis, anhydrobiosis, osmobiosis, or anoxybiosis, and specific mechanisms of desiccation protection have been proposed (e.g., specific vitrifying proteins, genetic damage suppressors) for tardigrades [5].

Overall, many of the cited natural examples have developed adaptive mechanisms to respond to low temperatures and to the different naturally occurring phase transitions of water (Figure S2). Therefore, a close relationship may be highlighted between the presence and/or the physical state of water within a defined biological system, the metabolic activity of the same system, and the surrounding environmental conditions. In this context, cryopreservation and lyophilization appear as adapted simplified models for studying natural metabolic activity modifications and the mechanisms of natural dormancy for example.

Indeed, both techniques may be used to experimentally reproduce the cryopreservation of microscopic worms or the cryptobiosis state of tardigrades [56,83]. As full control over the metabolic activity of complex biological lifeforms is not yet possible, the use of simple and controlled tools appears as an efficient study model. Furthermore, the appropriate use of biomimetic processes and approaches may be of great importance for gaining further insights and control over the key drivers of metabolic activity modulation in complex biological organisms. 

## 6. Origins and Evolution of Biological Lifeforms: Primordial Environment-Related Considerations

High diversity in biological lifeforms and entities exists in the modern biosphere, due to evolutionary drivers which have developed over the course of billions of years. Initially adopting the scale of micro-organisms in oceanic environments, primitive biological lifeforms were elevated to higher functions by the drive of natural selection [1,2,3,4,84,85]. Natural examples of dormancy demonstrate that, within the evolutionary development of biological organisms, several strategies have been perfected for conservation and protection against the effects of time, notably for survival or dissemination.

Within specific forms of dormancy or cryptobiosis, the metabolic activity may be limited to a fraction of its former level, yet “vitality” is still present in the organism as it may be restored to quasi-full function under the appropriate conditions [5,8]. Such observations may be indicative of the metabolic rates of primitive micro-organisms, progressively rising from infinitesimal levels to those which may currently be observed [1,2]. 

The high resistance of selected biological organisms to extreme environmental conditions as described herein (e.g., extremotolerance of tardigrades) may be attributed to the biological constituents of the same organisms. Altogether, the fact that tardigrades were shown to resist outer space conditions confirms the high acute resistance of biological structures and components to the most extreme environments [86,87,88]. Furthermore, long-term resistance of biological structures has been evidenced in specific protective environments (e.g., permafrost, amber; Figure S4) [89,90,91,92,93,94]. A prime example of such resistance to the effects of time has been the revival of bacterial spores in amber samples with a dated age of 40 million years [95].

## 7. Similarities between Industrial Biotechnology Conservation Processes and Natural Long-Term Preservation of Biological Organisms

When considering extremely low temperatures in the cryopreservation field and when tending toward the absolute zero in temperature, virtually all metabolic activity disappears. Therein, the potential of cryogenically frozen or otherwise preserved materials to achieve restoration of normal metabolic activity constitutes a central aspect of biological organism conservation. Cryopreservation and lyophilization techniques constitute appropriate examples for consideration of the environmental conditions and physical phase/state transitions necessary for reversible metabolic activity rate modifications. Indeed, while both controlled techniques are employed to directly modify the state and characteristics of water-containing samples, they imply the creation of adapted localized environmental conditions favoring the occurrence of specific transitions or changes [9,10,20]. Indeed, thermal energy may be subtracted or added to enable water freezing, melting, or sublimation/desorption, but the controlled action of the manipulator is directed on the localized environment, not on the sample itself (Table 2). The manipulator therefore adopts an indirect role in the process, by creating the appropriate environment, but is not an active and direct driver of said transformative process undergone by the sample. 

Despite enormous recent advances in the fields of regenerative medicine and genetic engineering, much progress remains to be made to better control and modulate the metabolic activities of complex biological organisms. Many groups have been searching for the means to artificially prolong biological lifespans by means of various technological manipulation (e.g., epigenetic rejuvenation, 3D printing for renewal of organs, integrated life support systems) [96,97]. However, the limits of current scientific and technical knowledge seem to allow only marginal lifespan prolongation in complex biological organisms. Based on the time necessary for evolutionary processes to effectively produce lasting effects with regard to longevity or metabolic rate adaptation mechanisms, it is probable that the development of artificial equivalents will remain out of human reach for decades to come. However, the study of well-known natural mechanisms of natural biological organism conservation under adverse environmental conditions yields tangible potential for better understanding and control over the key drivers of metabolic rate modulation. Such bioinspired considerations may then potentially be applied in optimized biotechnological development and manufacturing workflows, as well as in specific medico-therapeutic practices.

## 8. Conclusions

The controlled industrial processes of cryopreservation and lyophilization were parametrically described herein, providing insight into the careful equilibrium in localized environmental conditions required to reversibly stabilize biological samples. In particular, critical importance of the temperature modification rates of the preserved system was outlined, as they define the crystalline form and the phase transition modalities of water molecules within the samples of interest. Tangible examples were provided as regards the use of biotechnological conservation tools for optimization of cytotherapies and derivatives for cutaneous regenerative medicine under the Swiss progenitor cell transplantation program. Specifically, the case of the progenitor biological bandages was used to demonstrate that clinical successes with novel cell therapies are made possible notably by the preparation of a cryoconserved and off-the-freezer or off-the-shelf cell source. Similarities were then outlined between the described artificial environment modifications (e.g., in laboratories, industry) and some natural ecological niches known to favor metabolic rate modifications (e.g., cryptobiosis) in selected biological organisms (e.g., tardigrades). In particular, similarities were highlighted between natural and artificial cryoprotectants and lyoprotectants (e.g., glycerol, trehalose), confirming that many modern cryopreservation and lyophilization processes followed bioinspired approaches. Key examples of natural dormancy and adaptation capabilities to extreme environmental parameters then enabled a discussion about the emergence of early primordial biological lifeforms, from natural biotechnology and evolutionary points of view. Overall, the provided considerations confirmed the interest of further transposing natural conservation processes to controlled laboratory settings in view of gaining better bioinspired control and modulation capacities over the metabolic activities of complex biological organisms.

## Figures and Tables

**Table 1:** Process phases, involved methods, and physical processes/techniques of cell cryopreservation from pre-treatment to post-storage sample thawing. The involved risks and possible damage to the cells are presented for each step. This standard approach is used in the classical cytotherapeutic workflows investigated under the Swiss progenitor cell transplantation program (Figure 1) [15]. An illustrated workflow describing the various cryopreservation steps is presented in Figure S1.

Cryopreservation Process Phases	Involved Methods & Operator Manipulations	Physical Processes & Techniques	Risks & Possible Damage to the Cells
Pre-treatment	Formulation adaptation, solvent addition, bulk cell suspension dilution & dispensing in vials ^1^	Homogenization of samples in cell cryopreservation medium; vial filling	Direct cytotoxicity of cryopreservation medium; mechanical damage to the cells during processing
2. Freezing	Controlled-rate or flash lowering of sample temperature down to –80 °C or –196 °C ^2^	Sample water phase transition from liquid to solid, in crystalline and/or amorphous physical state ^3^	Formation of ice crystals incurring mechanical damages to the cells; solution effects or pH changes causing cytotoxicity
3. Cryogenic storage	Immersion in the vapor phase or liquid phase of liquid nitrogen and steady storage temperature maintenance	Cooling of the frozen samples down to cryogenic temperatures	Non-reversible slowing or arrest of cellular metabolic processes; undesired thawing due to equipment malfunction
4. Thawing	Retrieval of frozen samples from storage and rapid thawing at 37 °C ^4^	Sample water phase transition from solid to liquid	Slow sample warming and thawing causing mechanical stress to the cells or chemical cytotoxicity; solution effects or pH changes causing cytotoxicity

^1^ For the reported examples of therapeutic primary progenitor cells, optimal dilutions for efficient cryopreservation range between 10^6^ and 10^7^ cells per mL of cryopreservation solution. ^2^ Two-step freezing is usually performed using freezing devices (e.g., Mr Frosty, CoolCell) in ultra-low temperature freezers, while single-step freezing is usually performed in controlled-rate cryogenic freezers. Typical rates of cooling may be close to −1 °C/min for most mammalian cell suspensions, yet individual process adaptations should be performed for all biological materials to be preserved, in view of maximizing cellular viability. ^3^ Biological samples often present mixed crystalline-amorphous solid states. ^4^ Conservative best practices for cell cryopreservation generally prescribe slow freezing and rapid thawing rates [15]. Min, minutes.

**Table 2:** Process phases, involved methods, and physical processes/techniques of lyophilization for cell suspensions and cytotherapeutic derivatives from pre-treatment to storage. This standard approach is used in the cytotherapy-inspired workflows investigated under the Swiss progenitor cell transplantation program. Involved risks and possible damage to the cells or to the cytotherapeutic derivatives are presented for each step. An illustrated workflow describing the various lyophilization steps is presented in Figure S3. NA, non-applicable.

Lyophilization Process Phases	Involved Methods & Manipulations	Physical Processes & Techniques	Risks & Possible Damage to the Cells
Pre-treatment	Sample concentration, formulation adaptation, solvent removal, surface area optimization	Dialysis, filtration, evaporation, extraction, homogenization of the sample in lyophilization medium	Mechanical or thermal damage; chemical cytotoxicity
2. Wet Bulk Conditioning	Dispensing of the sample wet bulk in trays, recipients, or vials ^1^	Liquid dispensing and homogenization of the sample	Mechanical damage to the cells
3. Freezing	Slow controlled-rate cooling or rapid freezing below the triple point and eutectic point of the sample	Sample water phase transition from liquid to solid, creation of a crystalline and/or amorphous solid phase	Formation of ice crystals incurring mechanical damage to the cells; solution effects or pH changes causing cytotoxicity
4. Annealing	Temperature cycling	Reorganization of water in the solid phase	Formation of ice crystals incurring mechanical damage to the cells
5. Loading	Physical transfer of the batch to the freeze-dryer shelves ^2^	NA	NA
6. Primary Drying	Low-temperature energy provision by conduction, convection, or radiation	Sample unbound water phase transition from solid to gaseous state (sublimation)	Meltback or collapse of the sample; physical damage to the cells
7. Secondary Drying	High-temperature energy provision by conduction, convection, or radiation	Sample bound water desorption	Too much water removal; collapse of whole sample or of the cells
8. Unloading	Physical transfer of the sample batch from the freeze-dryer shelves ^3^	NA	NA
9. Dry Bulk Processing & Final Conditioning	Solid sample dry bulk processing or powder handling	Homogenization of the dry bulk	Mechanical damage to the cells
10. Storage	Transfer of the batch to a defined storage environment	NA	Degradation due to inappropriate conditioning or storage

^1^ Wet bulk dispensing may be performed in an intermediate conditioning material in view of lyophilization processing. ^2^ Loading may be performed prior to the freezing step in adapted freeze-dryers. ^3^ The final conditioning step may take place before unloading, with partial vacuum or inert gas stoppering of the vials.

## Data Availability

Not applicable.

## Supplementary Materials

The following supporting information can be downloaded at: www.mdpi.com/article/10.3390/biotech12010015/s1; Figure S1: Illustrative workflow for cytotherapy manufacture and cryopreservation; Figure S2: Illustrative examples of natural biological organism dormancy; Figure S3: Illustrative workflow for cytotherapy derivative lyophilization; Figure S4: Illustrative examples of long-term natural preservation of biological organisms or structures.
