# Supplementary material for: Industrial Biotechnology Conservation Processes: Similarities with Natural Long-Term Preservation of Biological Organisms
Source: BioTech (Basel). 2023 Jan 31;12(1):15. doi: 10.3390/biotech12010015 (PMC9944097; doi:10.3390/biotech12010015)
Supplement: Supplementary file 1 [file biotech-12-00015-s001.zip › biotech-2189849-supplementary.pdf]

BioTech

Supplementary Materials: Industrial Biotechnology Conservation Processes: Similarities with Natural Long-Term Preservation of Biological Organisms

Alexis Laurent, Corinne Scaletta, Philippe Abdel-Sayed, Wassim Raffoul, Nathalie Hirt-Burri and Lee Ann Applegate

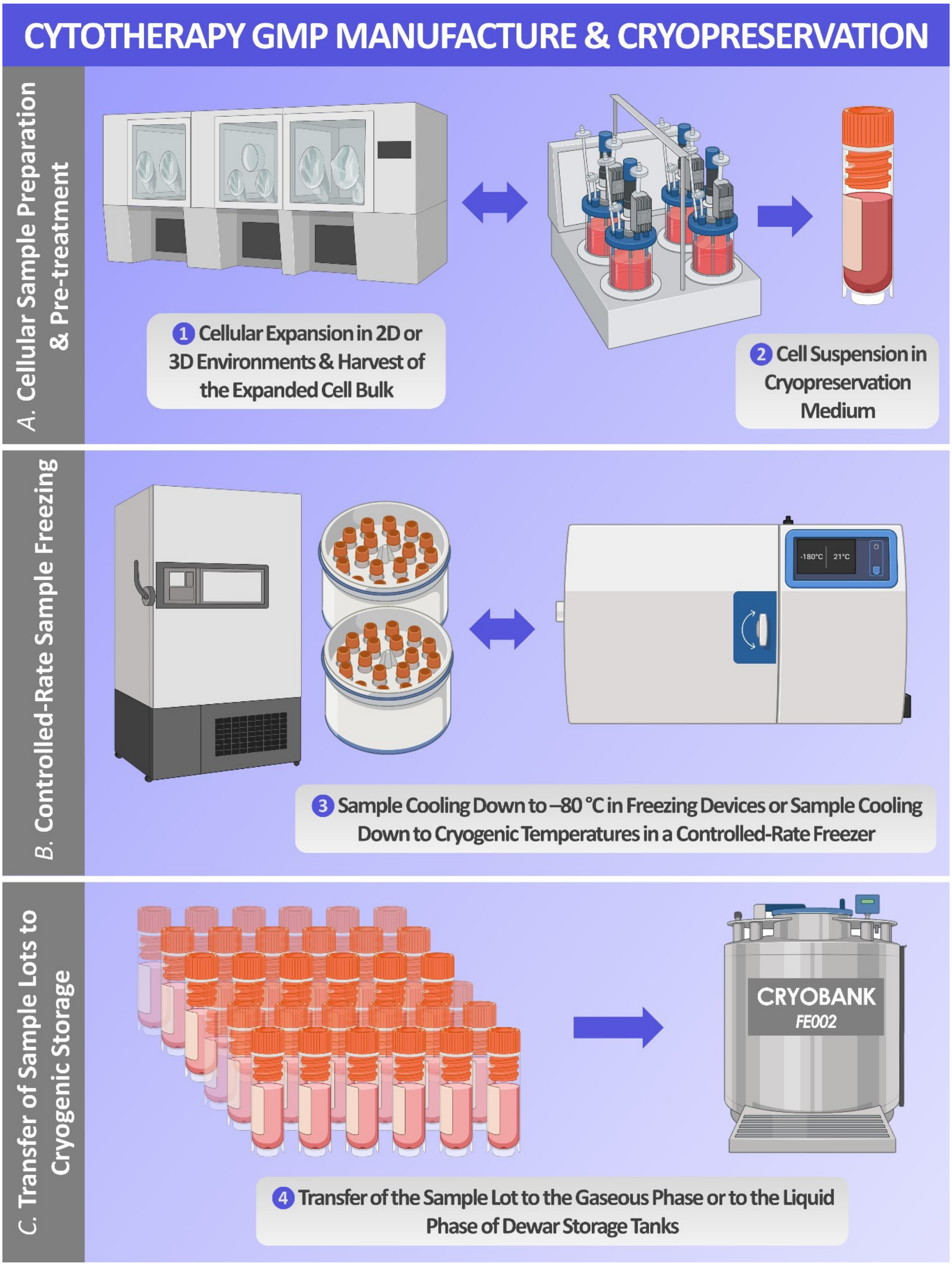

Figure S1. Illustrative workflow describing the sequential steps necessary for successful cytotherapeutic product cryopreservation [11,15]. Such processes are routinely used for classical cytotherapeutic approaches as investigated under the Swiss progenitor cell transplantation program. (**A**) Sample manufacturing in an appropriate in vitro system ❶, sample pre-treatment, and sample preparation phase, with formulation of the cell bulk suspension in an appropriate cryopreservation medium in adapted cryogenic vials ❷. (**B**) Controlled-rate sample freezing phase ❸, with two-step

or single-step cooling down to cryogenic temperatures (e.g., – 196 °C). (**C**) Transfer of the cryopreserved cell vial lot to long-term cryogenic storage vessels ❹, for subsequent cell banking or for further use in therapeutic preparations. GMP, good manufacturing practices.

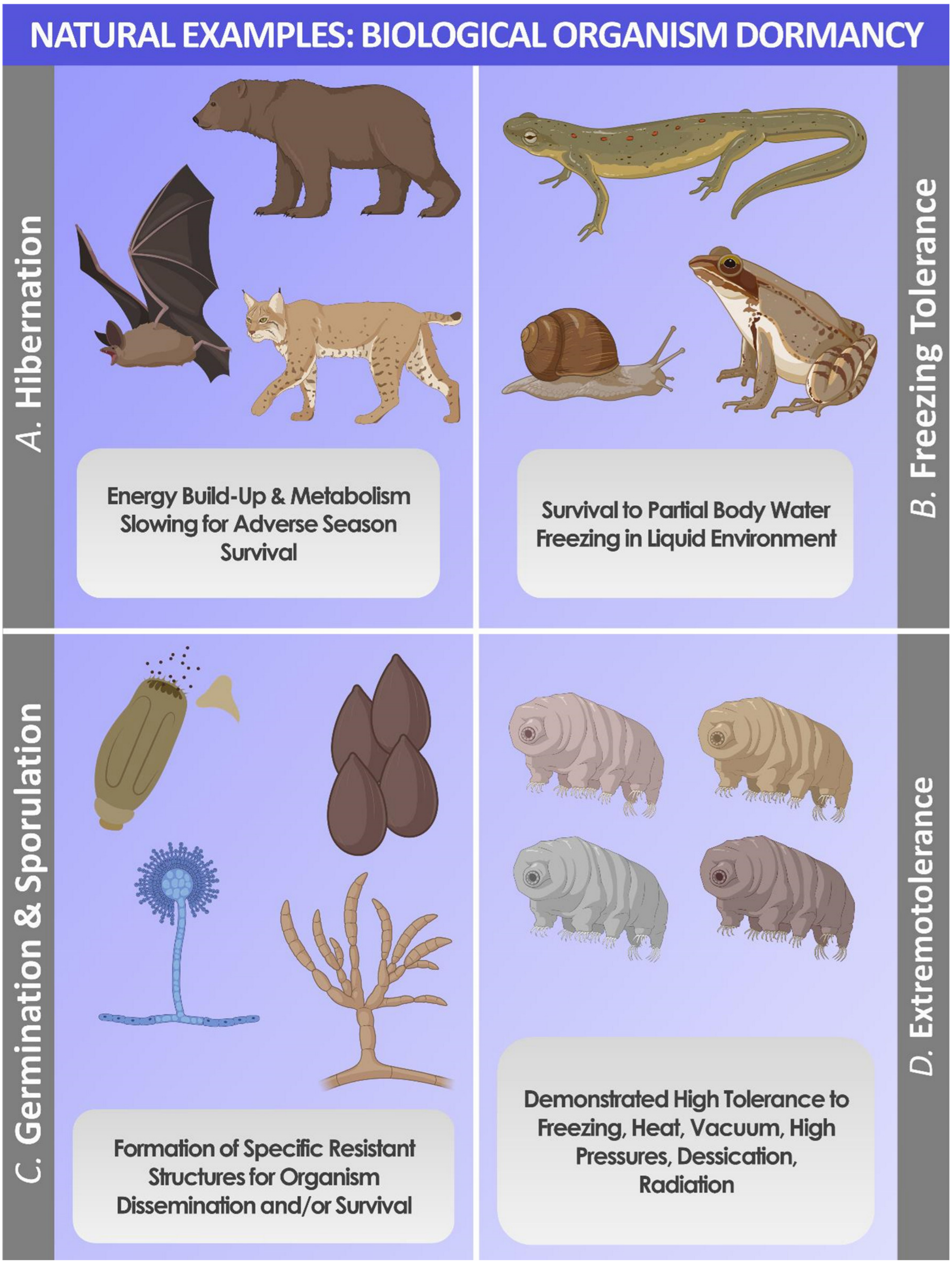

Figure S2. Illustrative examples of natural dormancy mechanisms, with some notable biological species using such mechanisms. (**A**) Hibernation is a periodic slowing of mammalian metabolic rates for winter survival, well-described for bears, lynx, and bats. (**B**) Freezing tolerance has been reported and demonstrated for several amphibian species such as salamanders and frogs, as well as snails. It is to note that only partial freezing of bodily water enables full functional

restoration upon eventual thawing. (**C**) Seeds and spores may be used by diverse biological organisms mainly for dissemination and survival purposes over prolonged time-periods. (**D**) Small multicellular organisms such as tardigrades were characterized as extremely robust and resistant to many environmental constraints, making them extremotolerant.

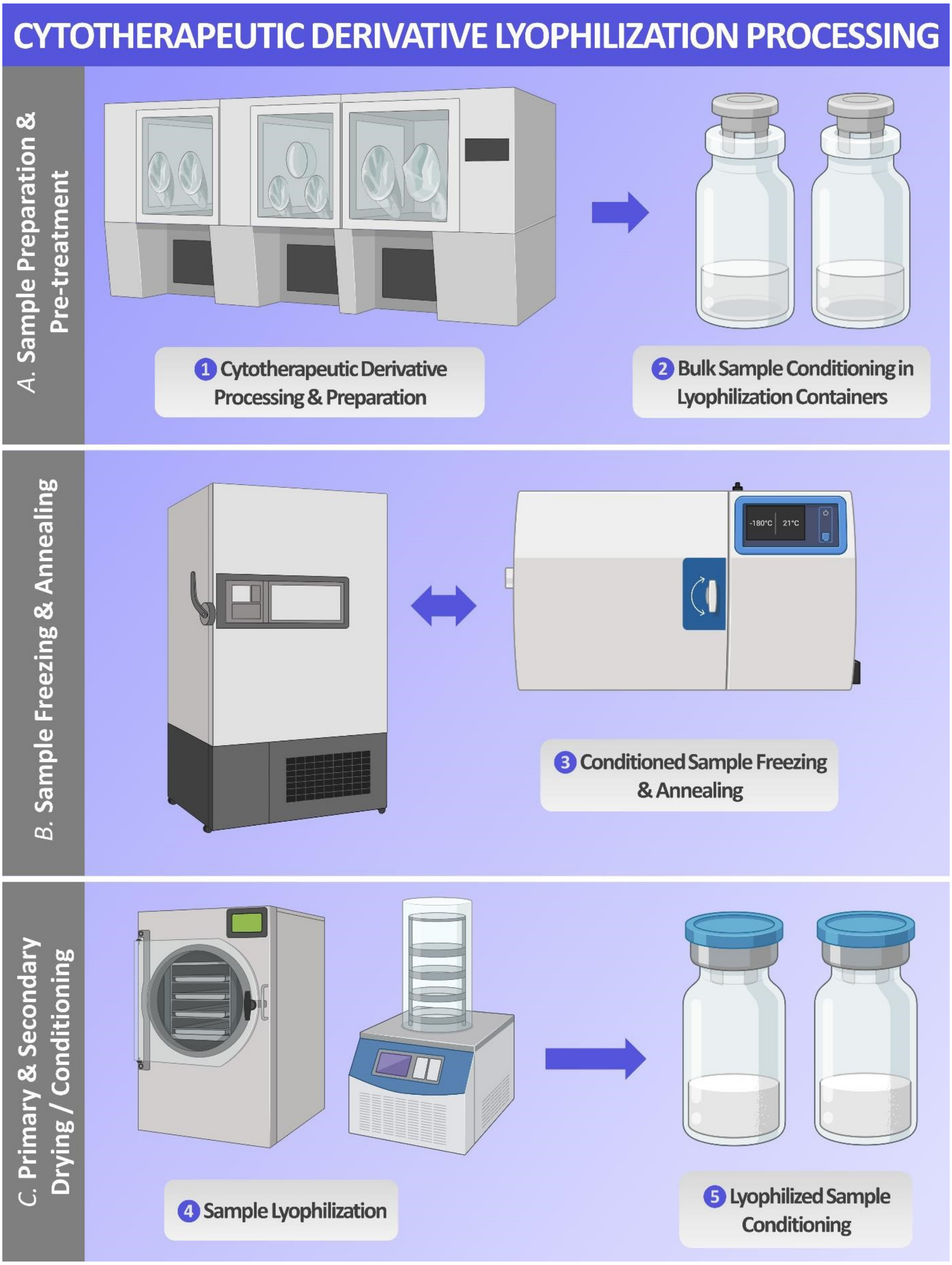

Figure S3. Illustrative workflow describing the sequential steps necessary for successful cytotherapeutic derivative lyophilization processing, as carried out under the Swiss progenitor cell transplantation program [12,65,72]. (**A**) Sample pre-treatment and preparation phase ❶, with formulation with an appropriate cryopreservation and lyopreservation medium in adapted lyophilization vials ❷. (**B**) Sample freezing and annealing ❸, for the obtention of a continuous and solid crystalline and/or amorphous phase. (**C**) Removal of sample water by means of two-step freeze-drying ❹, followed by final conditioning of the samples ❺.

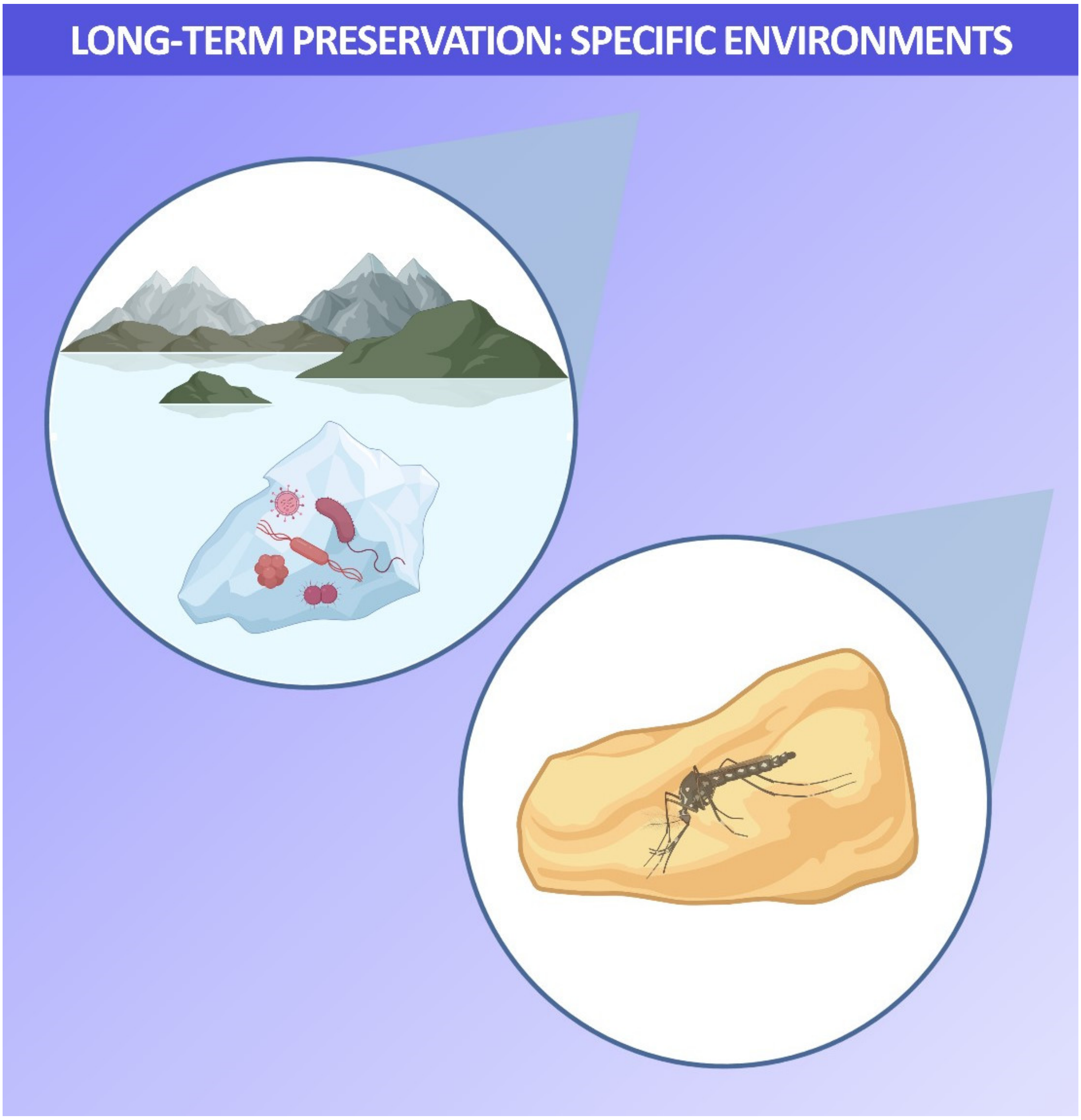

Figure S4. Schematic illustration of examples for two documented very long-term biological material preservation environments. The figure illustrates two specific local environments (e.g., glaciers/icebergs and permafrost, amber) enabling very long-term preservation of small organisms or biological structures [56,95].
